# A Case Report on *Pasteurella multocida* Peritoneal Dialysis-Associated Peritonitis: When Cats Think Medical Equipment Are Toys

**DOI:** 10.1155/2019/5150695

**Published:** 2019-12-09

**Authors:** Saeid Mirzai, Ahmad Oussama Rifai, Aron Tidrick, Qitan Huang, Justin Hale

**Affiliations:** ^1^Alabama College of Osteopathic Medicine, 445 Health Sciences Blvd, Dothan, AL 36303, USA; ^2^The Virtual Nephrologist, INC., PO Box 1750, Lynn Haven, FL 32444, USA

## Abstract

*Pasteurella multocida* is an aerobic gram-negative coccobacillus usually found in the oral cavities of most healthy cats and dogs as part of their natural oral flora. This zoonotic pathogen can cause a variety of infections in humans through bites, scratches, or licking. Infections range from less severe cases, such as infected animal bites and cellulitis, to more severe cases of pneumonia, septic arthritis, osteomyelitis, sepsis, and meningitis. However, the number of reported cases of peritoneal dialysis-associated peritonitis caused by *P. multocida* has been limited worldwide. Here, we report the case of a 59-year-old man undergoing continuous cycling peritoneal dialysis who developed *P. multocida* peritonitis, believed to be secondary to domestic cat exposure to dialysis equipment. Due to the increasing trend of pet ownership, patients maintained on peritoneal dialysis should be educated on the importance of strict hygiene and avoiding pet contact with the dialysis equipment, especially in bag exchange areas. Although the best means of preventing such infections is to avoid having pets at home, the positive psychological effects of pet ownership should also be considered. Thus, patients in such situations should be continuously educated and encouraged to be mindful of the importance of environmental hygiene.

## 1. Introduction

Aside from kidney transplantation, hemodialysis and peritoneal dialysis (PD) are two artificial renal replacement therapeutic methods used to treat patients with end-stage renal disease (ESRD). Both methods have their own limitations; thereby, one is not considered superior to the other. However, the application of PD is increasing, possibly owing to its lower cost and usage convenience since it can be performed at home and does not require travel to hospitals or satellite outpatient dialysis units [1].

Acute bacterial peritonitis in patients undergoing PD is a major cause of hospitalizations, transfer to hemodialysis, and catheter removal, presenting significant limitations to its utilization [2]. The major pathogens associated with peritoneal dialysis-associated peritonitis (PD-peritonitis) are the gram-positive cocci, *Staphylococcus aureus* and *S. epidermidis*, accounting for 42% of cases. These bacteria, originating from the skin, move through and along the dialysis catheter [3]. Gram-negative organisms are less commonly detected, accounting for 19% of cases [4]. A rare organism that has been shown to cause PD-peritonitis is *Pasteurella multocida,* with only 43 cases reported in the literature to date (Table 1).

*P. multocida* is an aerobic gram-negative coccobacillus found in the oral cavities of both domestic and wild animals [5]. It is part of their normal oropharyngeal flora, is spread through bites or scratches, and leads to various human diseases. Here, we report a case of PD-peritonitis caused by *P. multocida* in a patient with ESRD undergoing continuous cycling peritoneal dialysis (CCPD), believed to be caused by close contact with household cats.

## 2. Case Presentation

A 59-year-old Caucasian man with ESRD due to glomerulonephritis and failed kidney transplant who was undergoing CCPD for 7 months presented to the emergency department with periumbilical pain for several days. This was the first time the patient had experienced these symptoms since the initiation of CCPD. His medical history additionally included hypertension, valvular heart disease, degenerative arthritis, parathyroidectomy, and tobacco use. He had discontinued all of his immunosuppressive medications because his kidney transplant had been nonfunctional for several years.

Physical examination revealed a well-built, seemingly healthy man weighing 157 pounds with a blood pressure of 144/92 mmHg and a heart rate of 85 beats/min, without distress, and without jaundiced sclerae. He had clear lungs and systolic heart murmur. Abdominal examination revealed a soft abdomen with some fullness on the right lower quadrant proximal to his previous renal transplant, periumbilical tenderness, and intact exit site with no tunnel tenderness. He had active bowel sounds.

Laboratory testing revealed a white blood cell (WBC) count of 9.1 × 10^3^/*μ*L with 70% neutrophils, hemoglobin level of 12.9 g/dL, hematocrit count of 38.5%, and platelet count of 263 × 10^3^/*μ*L. Serum chemistry analysis showed the following levels: sodium 139 mmol/L, potassium 3.4 mmol/L, chloride 95 mmol/L, total carbon dioxide 28 mmol/L, glucose 127 mg/dL, blood urea nitrogen 31 mg/dL, and creatinine 12.46 mg/dL. The peritoneal fluid was colorless and appeared hazy. Initial analysis showed a WBC count of 4,470 cells/*μ*L with 85% polymorphonuclear cells and 15% mononuclear cells. Gram staining of the peritoneal fluid was negative for any organisms, only showing WBCs.

Based on the available information, the patient was diagnosed with PD-peritonitis, and empiric treatment with 1 g of vancomycin and 1 g of ceftazidime was administered intravenously and daily infusions initiated. The final diagnosis was made through peritoneal fluid culture that showed light growth of *P. multocida* after 3 days. The isolate was sensitive to ampicillin, ceftriaxone, ceftazidime, gentamicin, and ciprofloxacin. Therefore, antibiotics were adjusted according to the bacterial cultures, where intravenous ampicillin-sulbactam was started and then switched to oral amoxicillin-clavulanate for a total of 3 weeks. Patient had subsequent improvement in symptoms and laboratory results. He was able to continue PD successfully without any interruption or the need for catheter removal.

Upon taking further history, the patient reported having several cats at home, none of which were kittens. At night, he would begin his PD treatment and go into a different room to watch television. Therefore, the cats were assumed to play with the dialysis equipment or chew on the tubing, possibly leading to infection, although the patient did not recall noticing any fluid leakage from the tubing. Prior to discharge, the patient's dialysis technique was confirmed by direct observation, and aseptic technique was ensured. The patient additionally decided to place his cats outside the home at night before starting PD treatment. He did not have any more episodes of PD-peritonitis caused by *P. multocida* after changing his routine.

## 3. Discussion

*P. multocida* is an aerobic gram-negative coccobacillus found as one of the normal upper respiratory flora in various domestic animals, particularly cats and dogs. It was named after Louis Pasteur who first described the organism in 1880 [3]. The pathogen can lead to a wide variety of human conditions ranging from less severe cases such as infected animal bites and cellulitis to more severe cases of pneumonia, septic arthritis, osteomyelitis, sepsis, and meningitis. These severe conditions are more likely to occur in immunocompromised individuals due to the opportunistic nature of the organism [6]. PD-peritonitis secondary to *P. multocida* is very rare, with only 43 reported cases worldwide (Table 1).

The first case of PD-related peritonitis caused by *P. multocida* was reported by Paul and Rostand in Alabama in 1987 where the patient had acquired the infection from cat bites or scratches to the dialysis tubing [7]. Since then, the number of such cases reported in the literature is continuously increasing. This could be related to the growing population of ESRD patients, and the corresponding increase in PD as a home modality, indicating a correlation between home environment and medical therapy. According to the American Veterinary Medical Association, an increasing number of households are housing pets, with 30% of households owning two or more cats and 36% of households owning one or more dogs in 2012 [8]. This has led to an increasing risk of zoonotic transmitted infections over time.

The current literature on PD-peritonitis caused by *P. multocida* is summarized in Table 1, with the vast majority of cases acquired from cats and a small number from dogs. The prevalence of colonization with *P. multocida* has also been higher in cats (70–90%) than in dogs (50–66%), made worse by the sharper teeth found in cats [6]. Additionally, 20% of cats are found to have *Pasteurella* spp. on their claws [2]. The organism is spread through bites, scratches, and licking. The most common method which causes *P. multocida* PD-peritonitis is close contact of the cats with the dialysis equipment, including the tubing or bags; however, it can also spread through patients with pets who do not practice proper hygiene when preparing their treatment [4].

With regard to the types of PD, PD-peritonitis caused by *P. multocida *more commonly occurs in patients utilizing CCPD than in those using continuous ambulatory peritoneal dialysis (CAPD). This is possibly secondary to the length of tubing required or the prolonged amount of time the equipment is in contact with the environment as compared to CAPD. Cats may also enjoy the warmth of the heat plate and lay on the dialysis machine [9].

Although the majority of cases had direct contact with animals, there have been cases with no cat contact, or spontaneous cases in immunocompromised patients or those who have chronic diseases (such as malignancy, cirrhosis, diabetes, human immunodeficiency virus infection, chronic pulmonary disease, ESRD) [7]. Additionally, studies showing spontaneous oropharyngeal colonization in healthy livestock breeders indicated the possibility of colonization in patients who have housed pets for long periods of time [4]. Therefore, improper hygiene could lead to the spread of the organism from the patient's own saliva to their hands and dialysis equipment [5].

In terms of presentation, no specific clinical signs are typically present to differentiate *P. multocida* peritonitis from other etiologies [4]. Therefore, history taking is important in order to find out whether the patient has any pets and whether those pets have access to the dialysis equipment. Typical symptoms of peritonitis include low-to-moderate-grade fever, severe abdominal pain, and cloudy dialysate, in addition to occasional nausea and vomiting. Peripheral WBC count may be normal or show severe leukocytosis with increased polymorphonuclear leukocyte levels, and peritoneal dialysate WBC count is usually very high. Gram staining of the dialysate is usually negative, as it was in our patient [6]. Symptoms typically begin within 24 hours and improve within 48–96 hours after initiating the antibiotic therapy [5].

PD-peritonitis caused by *P. multocida* is usually susceptible to penicillin, which is considered the treatment of choice. Tetracyclines, cephalosporins (third- or later-generations), and fluoroquinolones are considered alternatives. Its susceptibility to aminoglycosides varies [7]. The typical duration of treatment is estimated to be 3 weeks according to the current literature, and the preferred route of administration is intraperitoneal [6]. PD-peritonitis prevention through proper hygiene and technique, or immediate treatment upon its occurrence, is critical due to the risk of possible peritoneal membrane failure because of severe and prolonged peritonitis. This is a major cause of PD termination and switching to hemodialysis [2]. Most cases of PD-peritonitis caused by *P. multocida* are resolved without catheter removal; however, the catheter should be removed if the patient continues to have cloudy effluent after an appropriate antibiotic treatment for 5 days, indicating refractory peritonitis [2].

*P. multocida* is a rare organism that causes PD-associated peritonitis in patients who keep pets, especially cats, in the environment where PD is performed. With the increasing trend of pet ownership, PD patients should be educated on the importance of strict hygiene and avoiding pet contact with dialysis equipment, especially in bag exchange areas [7]. The best method of preventing these infections is to avoid having pets at home by possibly relocating them with friends or family members while the patient is on dialysis. However, the positive psychological effects of pets on the patients' health should be considered [10]. Therefore, pet contact with dialysis equipment should be prevented and environmental hygiene maintained in patients who wish to have their pets at home. Additionally, patients should be continuously educated and encouraged to be cautious, especially during their switch from CAPD to CCPD [2].

## Figures and Tables

**Table 1:** Previous case reports on peritoneal dialysis peritonitis caused by *Pasteurella multocida.*

Case	Citation	Year of publication	Age (years)/sex	Etiology of ESRD	Animal exposure	Treatment
1	11	1987	55/F	Hypertension	Cat	Vancomycin, gentamicin
2	12	1991	55/M	Polyarteritis nodosa	Cat	Vancomycin, gentamicin, ciprofloxacin
3	13	1991	54/M	Hypertension	Cat	Vancomycin, gentamicin, cefazolin
4	14	1991	25/M	Alport syndrome	Cat	Gentamicin, cephradine
5	15	1996	75/M	Hypertension	Cat	Vancomycin, cefamandole
6	16	1996	42/F	Hypertension	Cat	Vancomycin, gentamicin, penicillin
7	17	1997	12/F	Focal segmental glomerulosclerosis	Cat	Cephapirin, gentamicin
8	18	1997	73/M	Chronic glomerulonephritis	Cat	Vancomycin, ceftazidime
9	19	1998	55/M	Polyarteritis nodosa	Cat	Vancomycin, gentamicin, ampicillin-sulbactam
10	20	1998	47/F	Type 1 diabetes mellitus	Cat	Piperacillin, ciprofloxacin
11	21	1999	49/M	IgA nephropathy	Cat	Cefazolin, tobramycin
12	22	1999	18/M	Interstitial nephritis	Cat	Ticarcillin, clindamycin
13	23	2000	69/F	—	—	—
14	23	2000	46/F	—	—	—
15	23	2000	64/M	—	—	—
16	24	2000	22/F	Medullary cystic kidney disease	Cat	Vancomycin, amikacin, ciprofloxacin
17	25	2002	24/F	Chronic pyelonephritis	Cat	Ciprofloxacin
18	26	2004	48/F	—	Cat	Cefazolin, gentamicin, ampicillin
19	7	2004	73/F	Autosomal dominant polycystic kidney disease	Cat	Vancomycin, gentamicin, Ciprofloxacin
20	27	2005	52/M	IgA nephropathy	Cat	Amikacin, cefazolin
21	28	2005	21/F	Congenital small kidneys	Cat	Gentamicin, ceftazidime
22	28	2005	58/M	Cyclosporine nephrotoxicity	Cat	Gentamicin, vancomycin
23	29	2006	46/F	—	Cat	Ceftazidime
24	30	2007	48/F	—	Dog	Cefazolin, gentamicin
25	31	2008	29/F	—	Cat	Cefazolin, tobramycin
26	6	2010	38/M	ANCA-related vasculitis	Cat	Ampicillin, levofloxacin
27	10	2010	36/F	Systemic lupus erythematosus, diabetes mellitus, hypertension	Cat	Ciprofloxacin
28	32	2010	58/M	Type 2 diabetes mellitus	Cat	Cefazolin, ceftazidime
29	2	2012	45/M	IgA nephropathy	Cat	Vancomycin, ceftazidime, levofloxacin
30	33	2012	57/M	Autosomal dominant polycystic kidney disease	Cat, dog	Vancomycin, ceftazidime
31	34	2013	49/M	Diabetes mellitus	Cat, dog	Vancomycin, tobramycin, cefazolin, ceftazidime, amoxicillin/clavulanate
32	4	2013	7/F	CKD secondary to prior cardiac arrest	Cat	Ampicillin
33	3	2014	62/F	Hypertension	Cat	Ciprofloxacin, trimethoprim-sulfamethoxazole, cephalothin
34	5	2014	25/F	—	Cat	Cefazolin, gentamicin
35	35	2015	28/F	Hypertension, congenital solitary kidney	Cat	Cefazolin, tobramycin, ceftazidime
36	35	2015	37/M	Diabetes mellitus	Cat	Cefazolin, tobramycin
37	35	2015	41/M	Diabetes mellitus, hypertension	Cat	Cefazolin, tobramycin
38	35	2015	51/F	Hypertension	Cat	Cefazolin, tobramycin, Amoxicillin-clavulanic acid
39	35	2015	37/F	Chronic interstitial nephropathy	Cat	Ceftriaxone, amoxicillin
40	35	2015	59/F	Diabetes mellitus	Cat	Cefazolin, tobramycin, ceftazidime
41	35	2015	69/F	Diabetes mellitus	Cat	Cefazolin, tobramycin, ceftazidime, amoxicillin/clavulanic acid
42	36	2017	72/M	Diabetes mellitus	Cat	Vancomycin, ceftazidime
43	9	2018	3/F	Congenital nephrotic syndrome	Cat	Cefazolin, piperacillin, cefdinir
44	Our case	2019	59/M	Glomerulonephritis, failed kidney transplant	Cat	Vancomycin, ceftazidime
